# Assessment and management of pain/nociception in patients with disorders of consciousness or locked-in syndrome: A narrative review

**DOI:** 10.3389/fnsys.2023.1112206

**Published:** 2023-03-20

**Authors:** Estelle A. C. Bonin, Nicolas Lejeune, Emilie Szymkowicz, Vincent Bonhomme, Charlotte Martial, Olivia Gosseries, Steven Laureys, Aurore Thibaut

**Affiliations:** ^1^Coma Science Group, GIGA-Consciousness Thematic Unit, GIGA-Research, Liège, Belgium; ^2^Centre du Cerveau^2^, Liège University Hospital, Liège, Belgium; ^3^Centre Hospitalier Neurologique (CHN) William Lennox, Saint-Luc Hospital Group, Ottignies-Louvain-la-Neuve, Belgium; ^4^Institute of Neuroscience, Université catholique de Louvain, Brussels, Belgium; ^5^Department of Anesthesia and Intensive Care Medicine, Liège University Hospital, Liège, Belgium; ^6^Anesthesia and Perioperative Neuroscience Laboratory, GIGA-Consciousness Thematic Unit, GIGA-Research, Liège, Belgium; ^7^Joint International Research Unit on Consciousness, CERVO Brain Research Centre, Centre Intégré Universitaire de Santé et Services Sociaux (CIUSS), University Laval, Québec City, QC, Canada

**Keywords:** pain, nociception, disorders of consciousness, locked-in syndrome, pain assessment, pain management, theories of pain

## Abstract

The assessment and management of pain and nociception is very challenging in patients unable to communicate functionally such as patients with disorders of consciousness (DoC) or in locked-in syndrome (LIS). In a clinical setting, the detection of signs of pain and nociception by the medical staff is therefore essential for the wellbeing and management of these patients. However, there is still a lot unknown and a lack of clear guidelines regarding the assessment, management and treatment of pain and nociception in these populations. The purpose of this narrative review is to examine the current knowledge regarding this issue by covering different topics such as: the neurophysiology of pain and nociception (in healthy subjects and patients), the source and impact of nociception and pain in DoC and LIS and, finally, the assessment and treatment of pain and nociception in these populations. In this review we will also give possible research directions that could help to improve the management of this specific population of severely brain damaged patients.

## 1. Introduction

Pain refers to an “*unpleasant sensory and emotional experience associated with, or resembling that associated with, actual or potential tissue damage*” (Raja et al., 2020). Integrating several dimensions (physiological, sensory, cognitive, and emotional aspects), pain is based on subjective experience and therefore, on conscious processing of the stimulus. Like any subjective experience, communication with the patient is the most appropriate way to assess it. In severely brain injured subjects, such as patients with disorders of consciousness (DoC) and locked-in syndrome (LIS), verbal communication is impaired but does not exclude the possibility that they experience pain. Even more, the absence of behavioral signs of consciousness does not preclude the patient to show (at least a minimum of) cortical activity preservation, suggesting partial preservation of consciousness and pain processing. Therefore, it is important not to neglect the assessment of pain and nociception in these patients with limited or no ability to communicate, regardless of the diagnosis. In the last years, clinicians tended to identify behavioral patterns related to conscious perception of pain, with important ethical and clinical implications in terms of diagnosis, prognosis, and treatment. Nonetheless, the absence of clinical signs of pain does not preclude a conscious (i.e., cortically mediated) pain experience nor a physiological impact of the nociceptive stimuli. Indeed, nociception refers to “*neuronal process allowing the encoding and processing of a noxious stimulus*” (Loeser and Treede, 2008) and while it does not require conscious perception of the stimulus, it leads to changes in the autonomic control of target organs (e.g., changes in heart rate, sweating, bronchial resistance to air flow, and pupil diameter) and behavioral responses (e.g., flexion withdrawal).

**FIGURE 1:**
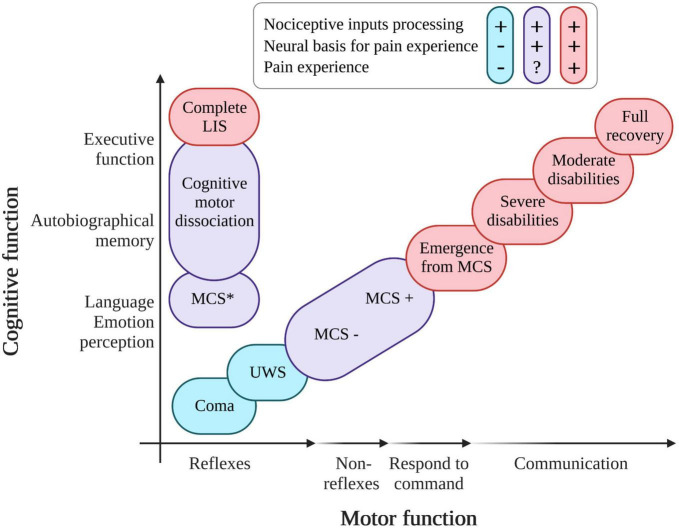
Variation in diagnosis of patients with pathological states of consciousness according to the level of recovery of cognitive and motor functions. UWS, unresponsive wakefulness syndrome; MCS, minimally conscious state; LIS, locked-in syndrome; in red, patients able to process nociceptive inputs and able to experience pain; in blue, patients processing nociceptive inputs but without evidence of pain experience; in purple, patients able to process nociceptive inputs and having the (probable) neural basis for pain experience (created with BioRender.com, based on Thibaut et al. (2019) and recent empirical literature).

Disorders of consciousness could be due to various traumatic (TBI) or non-traumatic (NTBI) brain injuries (e.g., strokes or anoxia). In the United States, 2.5 million people suffer from a TBI each year (288,000 hospitalizations and 56,800 deaths) and some of them will become unresponsive wakefulness syndrome (UWS) or minimally conscious state (MCS) patients (Capizzi et al., 2020). In the United States, the prevalence of patients with DoC (adults and children) is estimated between 4,000 and 25,000 for patients in UWS and between 112,000 to 280,000 for patients in MCS (The Multi-Society Task Force on PVS, 1994; Pisa et al., 2014). In Europe, the cases of UWS patients are estimated between 4,362 and 58,160 in a population of 727,000,000 (Ashwal, 2004). The current literature does not allow to give the prevalence of LIS patients on a global or national level. Due to the absence of or impaired communication in these patients, pain assessment and management is a major clinical and ethical issue. DoC include different clinical entities based on cognitive level and motor abilities (see Figure 1). For instance, a patient who shows signs of arousal characterized by eye opening periods but no signs of awareness (i.e., only reflexive movement and absence of cortical processes), will be categorized as in UWS (The Multi-Society Task Force on PVS, 1994; Laureys et al., 2010). Patients in a MCS show reproducible, responses without functional communication and have partial cortical processes. They are classified into two main groups based on language preservation: patients in MCS minus (MCS−) showing non-reflexive behaviors (Bruno et al., 2011b; Giacino et al., 2018), and MCS plus (MCS+) who have a preservation of higher level non-reflexive behavior and language abilities (Thibaut et al., 2020). The progress of neuroimaging techniques has also allowed the emergence of new terminologies to classify patients with “atypical” brain activity patterns such as: minimally conscious state star (MCS*, i.e., patients behaviorally diagnosed with UWS but preserving residual brain activity congruent with MCS diagnosis at rest or during a passive or active paradigm; Thibaut et al., 2021), covert cortical processing (CCP or higher-order cortex motor dissociation – HMD, i.e., patient behaviorally diagnosed in a coma, UWS, or MCS− but retaining brain activity upon passive task; Edlow et al., 2017), cognitive motor dissociation (CMD, i.e., patient behaviorally diagnosed in a coma, UWS, or MCS− but retaining brain activity upon active tasks; Schiff, 2015). Finally, when a patient regains functional communication or functional use of objects, he or she is considered to be emerging from MCS (Di Perri et al., 2016). LIS is not considered as a DoC but could be misdiagnosed with coma and UWS (Cistaro et al., 2018). This condition results from a lesion in the corticospinal and corticobulbar pathways of the brainstem due to vascular pathology, traumatic brain injury, masses in the ventral pons, infection, or demyelination (Das et al., 2021). LIS patients suffer from limbs, head, and face paralysis (i.e., quadriparesis) as well as verbalization/vocalization, breathing, and coordination impairments. LIS patients can communicate using eyelid blinks, vertical eye movements, or head movements (i.e., yes/no communication code or letter spelling communication; Lugo et al., 2015). EEG-based brain–computer interfaces also allow LIS patients to communicate through brain signals (Annen et al., 2020). So far, there has been limited scientific research on pain processing in LIS. However, according to a European survey of health professionals, 90% of them considered that patients in LIS are able to feel pain and need to be treated (Demertzi et al., 2014). For patients with DoC, according to a survey, 96% of health professionals believed that MCS patients can feel pain, compared to 56% believing that UWS patients can do so (Demertzi et al., 2009). Nevertheless, as explained above, some behaviorally unresponsive patients can still have a cortical activity preservation suggesting covert consciousness (and potentially a preservation of pain processing). It is therefore important to set up pain assessment tools and treatments that are independent of the clinical diagnosis to avoid mismanagement.

This manuscript aims at reviewing the current knowledge about the assessment and management of pain and nociception in patients with DoC and LIS. We will first give an overview of the physiology of pain and nociception. We will then look more specifically at the possible sources and impact of pain in these populations. Finally, we will describe the tools and treatments currently in place for the assessment and management of pain and nociception for this patient population. This narrative review is based on systematic reviews, meta-analyses, original articles, and case studies.

## 2. Neurophysiology of pain and nociception

In order to understand the difference between pain and nociception and to comprehend to what extent severely brain-damaged patients process nociceptive inputs and pain, we must first look at the neurophysiology of these two phenomena. When nociceptive stimulation occurs, following tissue damage for example, a signal will be generated at the endings of the nociceptive Aδ (i.e., thinly myelinated fibers responsible for faster signal transmission, mediate nociceptive inputs but also non-nociceptive heat and cold stimuli) and C-fibers (i.e., non-myelinated, polymodal nociceptors that are sensitive to chemical, mechanical, and thermal stimuli, including nociceptive hot – >48°C – and noxious cold – <11°C). These fibers synapse at the level of the dorsal horn with the second nociceptive neuron that continues its path into the spinothalamic tract to the thalamus (for the majority of the fibers). After the thalamus, the signal arrives at several cortical areas [i.e., the primary and secondary somatosensory cortex and the insula and the anterior cingulate cortex (ACC)]. All these cortical and subcortical structures and their connections form a network which is activated following a painful stimulus. In this review, we will use the term “pain-related neuromatrix” to refer to these brain regions activated following a noxious stimulus. However, it is important to note that this network is not only related to pain processing but has been identified more as a salience detection network (Melzack and Wall, 1965; Ingvar, 1999; Brown et al., 2018). This supports the multidimensional aspect of pain sensation, already highlighted by Melzack and Wall (1965), and subsequently confirmed by neuroimaging studies: pain is not the result of the activation of a single specific region but of a network (Mouraux et al., 2011; Mouraux and Iannetti, 2018). The pain-related neuromatrix can be divided in two parts comprising: (1) the lateral system, involved in the sensory dimension of nociceptive stimulus processing (i.e., localization, duration, and intensity) which includes the lateral thalamic nucleus, the primary somatosensory cortex (S1), the secondary somatosensory cortex (S2), and the insula and the posterior parietal cortex; and (2) the medial system, related to the affective dimension of pain and comprising the medial thalamic nucleus, the prefrontal cortex, the ACC, the posterior cingulate cortex (PCC), and the posterior medial cortex (Bushnell et al., 1999; Hofbauer et al., 2001). These regions (i.e., prefrontal cortex, thalamus, and ACC) are also part of the external and internal networks of consciousness which underlines the importance of these brain areas in the conscious perception of pain. The insula also has an important role in the affective processing of pain because it mediates the signal between the posterior insula (lateral system) and the rostral part of the ACC (medial system) (Coghill et al., 1999; Peyron et al., 2002). According to neuroimaging studies in healthy subjects during acute pain stimulation, the cortical and subcortical regions most involved in pain signal processing are S2, the insula and the ACC [for a review refer to Peyron et al. (2000)]. The use of hypnosis [i.e., a state of consciousness involving attentional focus and reduced peripheral attention, characterized by an increased ability to respond to suggestion (Elkins et al., 2015)] with analgesic suggestions leads to a decrease of brain activity in the ACC, and makes it possible to modulate the affective dimension of pain [for a review refer to Thompson (2019)]. The use of hypnosis has also been shown to be effective in relieving chronic pain. Indeed, in a 1997 study, a positive correlation between the perception of painful sensation and cerebral activity of the ACC was demonstrated (Rainville, 1997). Conversely, when the ACC and the insula are activated just before a nociceptive stimulation, an increase in pain perception is observed (Boly et al., 2007). Altogether, the literature shows that the ACC has an important role in modulating pain perception, notably by interacting with regions of the limbic system like the amygdala, the thalamus, and the hippocampus (Moriarty, 2011; Calabrò et al., 2017). These subcortical and limbic structures participate in the balance of activity between the fronto-temporo-parietal cortex (involved in consciousness; Demertzi et al., 2013; Di Perri et al., 2013, 2016) and the autonomic nervous system.

**FIGURE 2:**
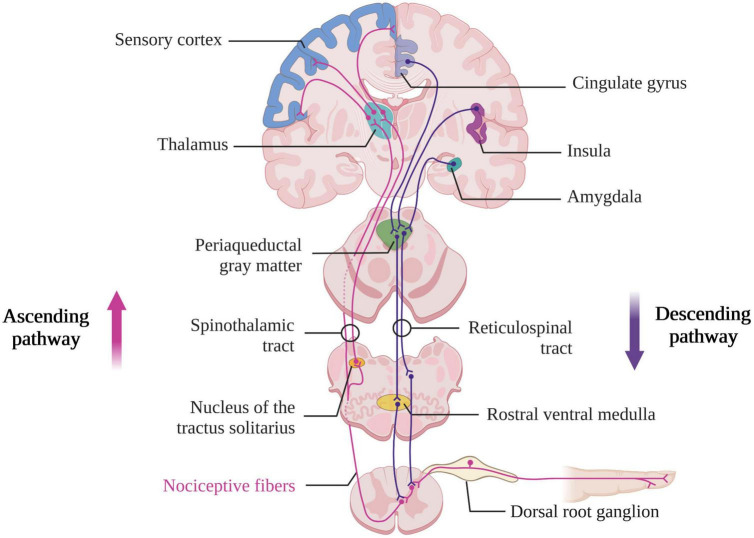
Ascending and descending nociceptive pathways (created with BioRender.com). Based on Brown et al. (2018).

The ascending pathways described above activate descending pathways responsible for modulating the transmission of peripheral information. Indeed, the descending pathways (cholinergic and serotonergic) to the dorsal horn of the spinal cord are sensitive to peripheral stimuli and not only to nociceptive stimuli. There are also tonic facilitation and inhibition phenomena that originate in the brainstem and respond to peripheral or non-peripheral stimuli (Dunckley, 2005). The activation of these descending pathways starts in the cortex (i.e., in the insula and ACC) and extends through the hypothalamus and the amygdala to finally be transmitted to the brainstem in the PAG, the nucleus of the tractus solitarius and the rostral ventral medulla (Brown et al., 2018). This will result in an inhibition of neurons from the superficial dorsal horn relaying information carried by C-fiber to the deep dorsal horn (Figure 2). The suppression of the C-fibers signal will then facilitate the transmission of sensory-discriminative information conducted by the A-fibers (Heinricher et al., 2009). The hypothalamus, the amygdala and the PAG are also responsible for behavioral changes related to acute pain stimulation (Veinante et al., 2013). An fMRI study of awake subjects undergoing acute thermal pain stimulation has shown a decrease of brain activity in the hypothalamus and amygdala as well as an increase brain activity in the lateral PAG (Robertson et al., 2022). The lateral PAG is involved in the selection of appropriate defensive behaviors in response to the nociceptive stimulus [i.e., increase in motor, autonomic, and endocrine activity, as well as alertness, and inhibitory control of this pain (Bandler et al., 2000)] and is regulated by a number of regions including the hypothalamus and the amygdala. A lesion in these different ascending and descending pathways therefore may lead to a dysfunction in the processing of nociceptive stimuli and pain control (i.e., which can result in the phenomenon of central sensitization).

The conscious perception of pain is supported by the activation of the regions evoked above and the functional connectivity between these different regions and the thalamus (Baars et al., 2003; Dehaene and Changeux, 2011). However, even if some of these regions are essential for pain sensation and sensibility, others will play a subtler role and their lesion may not always lead to any noticeable change in terms of pain perception. As explained above, the pain-related neuromatrix, although well established in the scientific literature, is still subject to debate, not least because the regions of this pain-related neuromatrix are more broadly involved in multimodal processing and not specific to pain processes (Mouraux et al., 2011; Mouraux and Iannetti, 2018). A recent opinion paper discussed the idea that pain perception may originate from the brainstem and not only from the cortex. The authors based this assumption in part on the fact that cortical stimulation of specific regions of the pain-related neuromatrix does not induce pain, unlike other sensory modalities (e.g., primary auditory or visual cortex stimulation evokes respectively sound and light). Few studies have nevertheless shown that the electrical stimulation of regions such as the parietal operculum, the posterior insula, and the ventral caudal nucleus of the thalamus could induce pain sensation (Lenz et al., 1993; Mazzola et al., 2006; Bergeron et al., 2021). However, a lesion of the insula does not make the sensation of pain disappear (Libet, 1973; Mazzola et al., 2006; Afif et al., 2008; Isnard et al., 2011). On the contrary, some patients who have suffered a cortical lesion (i.e., central post-stroke patients) experience an increased sensation of pain (Boivie et al., 1989; Andersen et al., 1995). As pain is necessary for survival, these authors suggest that its conscious perception must have been in place before the expansion of the cerebral cortex and therefore be located in the brainstem. Many brainstem nuclei are involved in nociceptive signal processing [for a review refer to Napadow et al. (2019)]. fMRI in humans show that, upon acute cutaneous or visceral stimulation, the PAG, nucleus cuneiformis, ventral tegmental area, substantia nigra, parabrachial complex, and dorsolateral pons regions of the brainstem become activated (Dunckley, 2005; Fairhurst et al., 2007; Sprenger et al., 2011). The spinal trigeminal nucleus located at the level of the medulla and caudal pons is activated during painful stimulation in the orofacial region (Nash et al., 2009). The brainstem also seems to be involved in the phenomenon of conditioned pain modulation. For instance, inhibition of orofacial pain *via* painful stimulation of another area (such as the leg) results in a reduction of the fMRI signal in the dorsal reticular nucleus, dorsolateral pons, and spinal trigeminal nucleus (Youssef et al., 2016). PAG and rostral ventral medulla also appear to be necessary for the temporal summation of pain in connection with the phenomenon of nociceptive wind-up [i.e., facilitation of neural discharges caused by repetitive stimulation of primary afferent C-fibers, involved in central sensitization (Mendell, 2022) in humans and animals with chronic pain (Van Oosterwijck et al., 2013; O’Brien et al., 2018)]. The study of the functionality of these different brainstem nuclei is challenging, especially in neuroimaging studies due to the location of these elongated and small cross-sectional nuclei, their proximity to cardiorespiratory noise sources. The role of these nuclei in pain processing has yet to be studied in LIS and DoC, however, these severely brain-injured patients may present cerebral deformations that make the analysis of robust neuroimaging data difficult.

## 3. Source and impact of pain and nociception in DoC and LIS

Due to their physical condition and the clinical environment in which they find themselves, patients with DoC and LIS may experience various types of nociceptive insults. For instance, acute nociceptive events can occur after injuries (e.g., fracture, wounds, and soft tissue/solid organ injuries) or during daily care (e.g., catheterization, surgery, or physiotherapy). If pain is present after those injuries, it will act as a protective and adaptive signal for the integrity of the body (Craig, 2003) whereas chronic pain loses the role of warning signal (Varrassi et al., 2010). Chronic pain is persistent and/or recurrent pain lasting for more than 3 months and can result in functional and emotional changes such as depression or anxiety (Grichnick and Ferrante, 1991; Merskey and Bogduk, 1994; Turk et al., 2011). It can be due to muscle contractions, pressure sores, peripheral nerve injury, pain network disruption leading to allodynia, central sensitization, neuropathic pain, or spastic paresis [for a review see Zasler et al. (2022)]. Central sensitization results from a dysfunction of the descending central control system and corresponds to an “*increased responsiveness of nociceptive neurons in the central nervous system to their normal or subthreshold afferent input*” (Loeser and Treede, 2008). Neuropathic pain is defined by the International Association for the Study of Pain as a type of “*pain caused by a lesion or disease of the somatosensory nervous system*” as opposed to nociceptive pain occurring following nociceptor stimulation. Neuropathic pain can be of central or peripherical origin depending on the lesion localization (Raja et al., 2020). At present, neuropathic pain may be identified by diagnostic testing [e.g., using questionnaires such as the DN4 (Bouhassira et al., 2005)], sensory testing coupled with self-report or neuroimaging (to locate the lesion). However, the use of questionnaires requires functional communication to express subjective experience and therefore cannot be used in patients with DoC. The difficulty of assessing this type of pain in DoC patients has so far not been addressed by any study. Regarding spastic paresis, a recent study showed that the majority (83%) of patients with DoC experience pain during physiotherapy sessions (Bonin et al., 2022b). This result can be explained in part by the high prevalence of spastic paresis in this population, ranging from 59 to 96% (Martens et al., 2017; Thibaut et al., 2021; Zhang et al., 2021; Bonin et al., 2022a,b). In addition to limiting patients’ motor responses, leading to misdiagnosis (Monti et al., 2010; Cruse et al., 2011), spastic paresis also appears to be related to the presence of nociception phenomena. Indeed, when looking at the scores of behavioral scales which respectively assess nociception and spastic paresis, it seems that both variables are positively correlated especially in wrist and finger muscles (Bonin et al., 2022a). This can greatly affect the patient’s ability to respond to commands and perform other motor-related tasks on which most of the Coma Recovery Scale-Revised [CRS-R, gold standard to assess the level of consciousness in DoC patients (Giacino et al., 2004)] items are based. Due to the fact that LIS patients are bedridden for long periods of time and therefore have limited mobility, they will develop pain mainly in the lower and upper limbs instead of the head, the back, and the abdomen (Bonin et al., 2022a). They will also be prone to develop spastic paresis, which can lead to persistent discomfort in the long run (Cairns and Stein, 2002; Pistoia et al., 2015). A 2022 survey investigated the presence and management of pain in this specific LIS population. The results highlighted that half of the LIS patients surveyed have pain but do not communicate about it and 92% of these patients suffer from chronic pain (Bonin et al., 2022c). Nociception may also have an influence on the autonomic nervous system, provoking an imbalance between sympathetic and parasympathetic activity (Lee et al., 2020). This can have hemodynamic consequences (e.g., increase in blood pressure, tachycardia, and increased heart rate variability), or influence other target organs of the autonomic nervous system (pupils and their diameter, sweat glands, and skin conductance) [for a review see Lee et al. (2020)]. Although no studies exist on this topic in patients with DoC or LIS, it can be assumed that repetitive and/or long-term autonomic nervous system imbalance due to acute or chronic nociception or pain could have consequences for the patient’s wellbeing, and could lead to systemic complications (Leo et al., 2016). For instance, it has been shown that, in moderate to severe traumatic brain injured (TBI) patients, autonomic nervous system dysfunction is correlated with an increase in morbidity (Purkayastha et al., 2019).

The perception of pain can vary according to different factors. Numerous studies carried out over the last few decades have revealed gender differences in terms of prevalence, perception and treatment of pain [for a review see Pieretti et al. (2016)]. Although women seem to report signs of pain more often than men, experimental studies on healthy subjects show mix results depending on the type of stimulation (i.e., mechanical, electrical, thermal, ischemic, and chemical) and the type of investigated parameter [e.g., duration and intensity of the pain sensation or pain tolerance or sensitivity (Labus et al., 2008; Racine et al., 2012)]. However, to our knowledge, no study has investigated gender differences in terms of pain perception in DoC and LIS patients. In LIS patients, the position (lying/sitting) can increase or decrease the pain sensation, depending on each individual (Bonin et al., 2022c). Pain has a direct influence on patients’ quality of life such as sleep quality, cognitive abilities, and emotion (Bonin et al., 2022c). Previous studies showed that the majority of patients with chronic pain have sleep disorders and that poor sleep quality can increase pain perception (O’Brien et al., 2011; Rousseau et al., 2015; Frohnhofen, 2018; Bonin et al., 2022c). In addition, the impact of pain on sleep quality can alter the level of arousal, as well as motivation in patients with DoC or in LIS. In this way, their ability to express signs of consciousness may be impeded, hence compromising the clinical diagnosis (Lanzillo et al., 2016; Estraneo et al., 2022). Consequently, the implementation of treatment to alleviate pain could have a positive impact on sleep quality and allow an improvement in the patients’ level of arousal/vigilance during clinical examinations. Deleterious effects of pain on cognitive abilities (i.e., increase of tiredness and mood swings, and decrease of memory and concentration) and emotional regulation has been observed in patients with LIS (Bonin et al., 2022c). Other surveys found that some patients in LIS claim experiencing anxiety, depression or suicidal thoughts (Bergés et al., 2007; Rousseau et al., 2015). Furthermore, a past study has found an anti-correlated relationship between perceived pain and life satisfaction (Skevington, 1998; León-Carrión et al., 2002). Several variables can be related to the decrease of life satisfaction in patients with LIS such as the loss of mobility during recreational activities or language impairment/speech production (as communication seems to play a key role in the preservation of the quality of life in those patients) (Bruno et al., 2011a; Demertzi et al., 2014). These results underline the importance of identifying the sources of potential pain by using appropriate tools, to propose patient-tailored management.

## 4. Pain and nociception assessment and management in DoC and LIS

### 4.1. Assessments

#### 4.1.1. Behavioral scales

There are many behavioral scales allowing the assessment of pain in non-communicative patients, such as the Neonatal Infant Pain Scales (NIPS; Lawrence et al., 1993), the Faces, Legs, Activity, Cry, Consolability pain scale (FLACC; Merkel et al., 1997) or the Children and Infants Post-operative Pain Scale (CHIPPS; Buttner and Finke, 2000) that assess pain in newborn, infants or adolescent. Other scales include the Pain Assessment In Dementia Scale for patient with dementia (PAINAD; Warden et al., 2003) and the Checklist of Non-verbal Pain Indicator to assess pain in cognitively impaired older adults (CNPI; Feldt, 2000). None of these scales are specific to severely brain-injured patients with DoC and LIS. The Nociception Coma Scale (NCS) has been developed to fill this gap (Schnakers et al., 2010) and consists in four subscales assessing motor, verbal and visual responses, as well as facial expression. It allows to disentangle reflex (e.g., groaning or oral reflex movements) from higher-level behaviors (e.g., pain localization and cry or intelligible verbalization). The visual subscale is the only subscale of the NCS that does not show significant changes between a noxious and a non-noxious condition. As its absence does not alter the sensitivity of the assessment, it was eventually removed to give the Nociception Coma Scale-Revised [NCS-R; Chatelle et al. (2012) total score ranging from 0 to 9]. The NCS-R is sensitive to the level of consciousness, with patients in MCS having higher NCS scores than patients in UWS, and allows the distinction between noxious and non-noxious stimulation (Chatelle et al., 2012, 2014b,^2018^). A neuroimaging study in DoC using labeled Fluoro-Deoxy-Glucose (FDG)-PET found a positive correlation between brain activity in the ACC and NCS-R scores, suggesting that these scores are related to a cortical processing of pain (Chatelle et al., 2014a). This scale might also give an indication on the probability of recovering consciousness. Indeed, in a recent study, 76% of the patients in UWS who evolved to MCS showed significant behavioral changes at the NCS-R and NCS 1 week before the new diagnosis. Threshold for prediction has been determined for the NCS-R and the NCS and showed high predictive accuracies (Cortese et al., 2021). However, the NCS provides a better classification of patients likely to evolved to MCS than the NCS-R due to the presence of the visual scale (i.e., visual pursuit and fixation are among the first signs of consciousness observed in patient recovering from a UWS). In clinical practice, mechanical stimulation (i.e., pressure on the nail) is used to perform the assessment by the NCS-R. One study highlighted that the pain threshold following mechanical stimulation (i.e., pressure on the nailbed with an algometer) was lower in patients with DoC than in healthy subjects (Sattin et al., 2017). However, this stimulation technique has very high inter-rater variability. If not performed using an algometer, it allows limited control of the stimulus intensity. Another study conducted in 2019 showed that the use of personalized stimuli, determined on a case-by-case basis by the clinical team during patient mobilizations, resulted in higher scores on the NCS-R compared to standardized stimuli (Formisano et al., 2020). This could allow a case-by-case assessment depending on the patient, particularly in prolonged DoC patients suffering from pain during mobilization at the moment of care (Bonin et al., 2020, 2022b). The NCS-R is a relevant behavioral tool for pain assessment in non-communicative brain-damaged patients [for a review on psychometric values refer to Vink et al. (2017)], as NCS-R scores appear to be related to cortical processing of pain and nociception.

A 2012 study tried to determine an NCS-R cut-off score allowing discrimination between noxious and non-noxious stimulation, but the result was not confirmed in a later study (Chatelle et al., 2012, 2018). Chatelle et al.’s (2018) study determined an NCS-R cut-off score of 2 as being related to nociception (i.e., obtainable by reflex behaviors such as flexion withdrawal or oral reflex movement). Nevertheless, the presence of these reflex behaviors does not necessarily imply a conscious perception of pain. Finally, a recent study based on neuroimaging data (i.e., FDG-PET), determined a conservative NCS-R cut-off score of 5 as being specific to a cortical processing of pain and allowing the detection of covert consciousness (e.g., MCS*). The study highlights brain metabolism differences between “FDG-PET confirmed UWS” patients (i.e., patient diagnosed as UWS with the CRS-R and with a global hypometabolism), patients with potential pain (i.e., UWS and MCS patients with NCS-R score ≥5) and healthy subjects at both global and regional levels (i.e., left insula – involved in the processing of the sensory and affective dimension of pain) (Bonin et al., 2020). Although this cut-off score is very conservative, it has a low sensitivity, which means that patients with a score of less than 5 should not be overlooked as they may still suffer and need appropriate treatment.

Studies involving nurses working with DoC patients confirm the ease of use and clinical relevance of this scale in assessing signs of pain in this population (Vink et al., 2014; Poulsen et al., 2019). Nonetheless, respondents considered that the use of a cut-off score underestimates the number of patients in pain and suggested that the use of physiological measures to complement the behavioral assessment should be favored (Poulsen et al., 2019). In cases of severe spastic paresis or intubation/anarthria, the facial expression subscale of the NCS-R is the only subscale on which the clinician can rely (Garuti et al., 2014; Thibaut et al., 2015). However, some facial expressions assessed by the NCS-R such as groaning or grimacing are not only associated with nociception but can be signs of agitation (Corrigan, 1989; Bogner et al., 2015). A study investigating the clinical relevance of the NCS-R in tracheostomized DoC patients showed that both the total score and the verbal subscore of the scale were decreased in DoC patients with tracheostomy compared to DoC patients without tracheostomy (Lejeune et al., 2020). However, the presence of a tracheostomy had no impact on the sensitivity and specificity of the cut-off score of 2. The authors recommend that the NCS-R should still be used in these patients but that the presence of a tracheostomy should be specified and taken into account in the assessment. Together, these studies confirm the experimental and clinical utility of the NCS-R in the assessment of pain in patients with DoC (Chatelle et al., 2016). As a corollary, these studies emphasize the need for clear guidelines regarding its use.

For appropriate daily management of pain, it appears that the NCS-R is not sufficient alone. The clinical assessment of pain should be based on a multi-modal approach that also considers (neuro)physiological markers wherever possible. Behavioral scales also involving physiological markers have recently been created, such as the Pain Assessment Scale (PAS), devoted to the assessment of patients with acquired brain injuries (Poulsen et al., 2016). It consists of 27 items, divided into four sections, and assessing physiological/autonomic responses, body language, verbal communication, and behavior during potentially painful manipulations. Preliminary results from this study show that half of the assessed items (7 of which were physiological markers) obtained very good inter-rater agreement, suggesting that some of them could be included in a new pain scale. Then, based on these results, the Brain Injury Nociception Assessment Measure (BINAM) was developed to measure nociception intensity in patients with severe brain injury who are unable to communicate (Whyte et al., 2020; for a comparison of the different scales refer to Supplementary Table 1). It consists of 10 items assessing both behavioral (e.g., facial expression and presence of tears) and physiological (e.g., respiration rate and skin temperature) parameters related to the processing of a nociceptive stimulus. The scores are independent of the diagnosis or state of agitation of the patients and appear to be sensitive to pain-inducing conditions (e.g., physiotherapy) as well as analgesic treatments (Whyte et al., 2020). However, studies are still needed to validate its clinical utility. The NCS-R is in fact the only behavioral scale recommended by the guidelines of the American Academy of Neurology (Giacino et al., 2018).

Regarding patients in LIS, in most cases, communication through eye movements or the use of Brain Computer Interface (BCI) technology is possible. Therefore, pain is assessed using communication codes (e.g., yes/no communication code *via* blinking, or alphabetic code) or/and visual analogue scale (VAS) ranging from 0 to 10 (0 = no pain, 10 = most severe pain). In spite of these systems, some patients in LIS do not communicate about their pain. In a 2022 survey, 52% of the painful patients declare that they do not inform the clinical teams about their pain (Bonin et al., 2022c) and only 28% of them use a communication code to communicate their pain. Other means of communication such as crying or wincing were also used by the patients but might be confounded with reflexive behavior. These results demonstrate how important it is for the clinical team to assess the signs of acute and chronic pain on a daily basis, through the use of communication codes or BCI techniques (Annen et al., 2020).

#### 4.1.2. Neuroimaging

**FIGURE 3:**
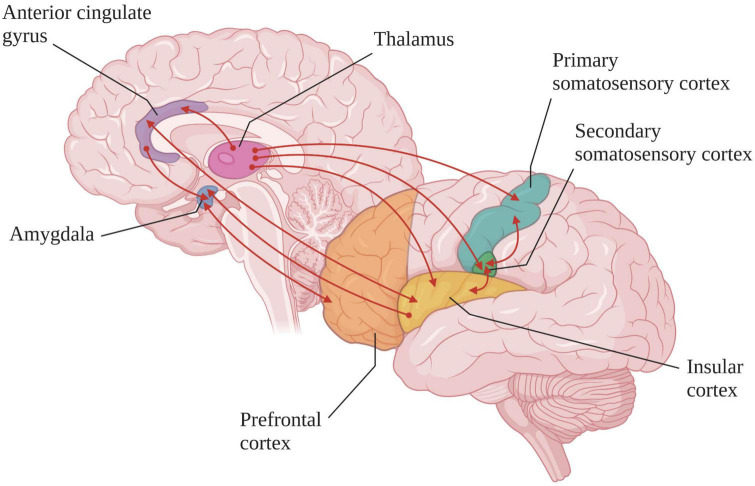
Cortical and subcortical regions involved in the pain-related neuromatrix (created with BioRender.com). In healthy subjects and MCS or LIS patients the functional connections (red lines) are preserved whereas in “true” UWS patients the somatosensory cortex is activated in isolation [based on Bouhassira et al. (2005) and Bagnato et al. (2021)].

Patients with DoC suffer from fronto-parietal network activity and functional connectivity dysfunction, which could lead to a disturbance in pain and nociception processing. However, neuroimaging studies carried out in this population have shown that some brain regions are preserved (see Figure 3; Laureys et al., 2002; Boly et al., 2008). Indeed, when nociceptive electrical stimulation (i.e., stimulation intensity judged as highly unpleasant to painful in healthy subject) is administered to MCS patients, the cortical activation pattern is close to that observed in healthy subjects and LIS patients, especially in the secondary somatosensory cortex, the ACC and the insula (Boly et al., 2008). Functional connectivity within the pain-related neuromatrix is also preserved in these patients (Kupers et al., 2005). Although if the activation of the pain-related neuromatrix is more lateralized and with a smaller spatial range, these results suggest that patients in MCS are able to consciously process pain. In contrast, in UWS patients, nociceptive electrical stimulation results in an isolated activation of the primary somatosensory cortex with an absence of functional connectivity with other regions involved in pain (Laureys et al., 2002). However, in 2003, a Positron Emission Tomography-H_2_^15^O activation study, tracking regional cerebral blood flow response to an external stimulus or task was performed in seven patients in UWS. After a nociceptive electrical stimulation, an increase in cerebral blood flow in the primary and secondary somatosensory cortices and in the ipsilateral posterior insula was observed (Kassubek et al., 2003). Another study using fMRI showed that during nociceptive electrical stimulation, 50% of patients in UWS have activation of the sensory network and 30% an activation of the affective network (Markl et al., 2013). These results suggest that residues of the pain processing network remain active in some patients considered as “unconscious” from a behavioral point of view. In these cases, the re-assessment of the diagnosis should be considered in patients who do not fit the criteria of a “real” UWS but rather those of MCS*.

#### 4.1.3. Neurophysiology

Event-related potentials (ERPs) evaluates the integrity of the central and peripheral sensory pathways within the nervous system (Koenig and Kaplan, 2015). For instance, somatosensory evoked potentials (SEPs), brainstem auditory evoked potentials (BAEPs), and visual evoked potentials (VEPs) are used as prognostics tools in acute comatose patients, with the absence of ERPs at the cortical level being associated to a poor outcome (André-Obadia et al., 2018; Rollnik, 2019; Bagnato et al., 2021). It is possible to detect SEP at the cortical level in comatose patients following a noxious stimulation. Some studies have highlighted that the presence of SEPs following median nerve electrical stimulation may appear to be predictive of a good neurological outcome in comatose patients, characterized by a score of 1 (i.e., conscious with normal functions) or 2 (i.e., conscious with moderate disability) at the Glasgow-Pittsburgh Cerebral Performance Categories (Zanatta et al., 2012, 2015; Markl et al., 2013). However, SEPs support the assessment of the functioning of the somatosensory system which, unlike the other ERPs mentioned above, includes several modalities. Indeed, SEPs assesses both Aδ (i.e., encoding thermal nociceptive and non-nociceptive inputs) and Aβ fiber pathways (i.e., encoding sensitivity to pressure or vibration), and therefore reflect the processing of the stimulus by the both spinothalamic and lemniscal pathway. In contrast, laser evoked potentials (LEPs) are specifically used to study nociceptive signal processing by looking at the integrity of the spinothalamic pathway. They are intimately linked to the stimulation of Aδ and C nociceptive fibers (i.e., encoding sensitivity to non-noxious hot and cold, as well as pain) (Treede et al., 2003). Stimulation of Aδ and C fibers can be done separately depending on the method used. One study showed that, in some UWS patients, it was possible to observe LEPs at the cortical level during C-fiber stimulation even in the absence of LEPs related to Aδ-fiber stimulation (Naro et al., 2015). On the other hand, the reverse does not seem to be achievable, which underlines the importance of including the C-fiber stimulation in the assessment of LEPs in UWS patients. However, the results of this study must be interpreted with caution as selective C-fiber stimulation in DoC patients is very difficult to achieve without a strictly temperature-controlled laser or without the patient’s cooperation in reporting his or her sensations. The LEPs recording consists of an early component N1, a late vertex components N2–P2 and an endogenous component P3 (only evoked during attentional tasks; Treede et al., 2003). Several studies have highlighted the presence of the N1 and N2-P2 complex at the cortical level in some patients with UWS (de Tommaso et al., 2013, 2015). However, cortical reactivity to nociceptive stimuli (characterized by prolonged N2 and P2 latencies) was decreased in these patients compared to healthy subjects, suggesting impaired functional connectivity. A case study also found a significant relationship between N2-P2 amplitude and the CRS-R scores in DoC patients. In this study, N1 and N2-P2 complexes were observed in MCS patients and only in one UWS patient but with a high CRS-R and NCS-R score (De Salvo et al., 2015). Coupled with SEPs, LEPs also detects potential lesions of the spinothalamic pathways in the dorsal brainstem. A lesion in this region impairs LEPs response while keeping SEPs intact (Treede et al., 2003). In the study of de Tommaso et al. (2015), the authors also studied the responses to auditory, visual and electrical (non-noxious) stimulation and found negative-positive complexes similar to the responses obtained after noxious laser stimulation. This confirms that a noxious stimulus will activate the same brain regions as another type of sensory stimulus (de Tommaso et al., 2015). Moreover, the presence of LEPs seems to be associated with cortical arousal in response to salient nociceptive stimuli (i.e., potentially dangerous stimulus) rather than with conscious pain processing.

#### 4.1.4. Physiological markers

Another way to study nociception that is widely implemented in the clinic is the measurement of physiological markers. Numerous brain areas forming the pain-related neuromatrix are also involved in modulating autonomic nervous system activity by integrating nociceptive and visceral information in the dorsal horn, insular cortex, amygdala, nucleus of the tractus solitarius, PAG, ACC, thalamus, hypothalamus, and *via* the neurons of Lamina 1 in the dorsal horn (Benarroch, 2001, 2006; Leone et al., 2006; Hohenschurz-Schmidt et al., 2020). This highly specialized organization of nociceptive information in these brain areas may play a major role in the development of an autonomic, affective, and emotional responses to pain (Benarroch, 2001, 2006; Leone et al., 2006; Cortelli et al., 2013; Hohenschurz-Schmidt et al., 2020). Processing of the nociceptive signal leads to homeostatic changes like heart rate variability (HRV), skin conductance or pupillary dilatation reflex (PDR). These physiological markers can therefore be a good index of the autonomic nervous system reactivity following nociceptive stimulation. Nociceptive pathways also have bidirectional interaction with the neuro-endocrine immune system, leading to a humoral response with potential consequences on recovery such as chronic pain (i.e., neuropathic or inflammatory pain) or stress response to surgery. Indeed, in addition to being sensitive to chemical, thermal, and mechanical stimuli, nociceptors are also able to detect immune mediators (e.g., cytokines, lipids, and grow factors) as well as certain pathogens (Basbaum et al., 2009; Chiu et al., 2012, 2016). Following the activation of nociceptors by these different agents, the signal is transmitted to the central nervous system to induce pain [e.g., microglia and T cells are involved in central sensitization (Ji et al., 2014)]. In response to this stimulation, the nociceptors will release neuropeptides that regulate the immune response [for a review refer to Baral et al. (2019)]. The study of interactions between pain and immune pathways is still poorly developed in DoC and LIS patients. A better understanding of these mechanisms in these specific patient populations could lead to new treatments for chronic neuropathic or inflammatory pain.

The most studied physiological marker to evaluate pain in DoC is HRV, which corresponds to changes in the time interval between successive heartbeats. It can provide information about the sympathetic/parasympathetic balance. This is a non-invasive measurement using an electrocardiographic (ECG) recording which takes only a few minutes (Palma and Benarroch, 2014; Riganello et al., 2018). The calculation is based on the interval between the R peaks of the QRS complex extracted from the ECG signal and analysis can be performed in the time or frequency-domain or using non-linear methods [for a review see Laborde et al. (2017)]. Numerous studies in healthy subjects as well as in different patients populations have demonstrated the link between pain/nociception and HRV [for a review refer to Forte et al. (2022)]. The changes in HRV observed during nociceptive stimulation, are not dependent on the method of stimulation since variations in heart rate have been observed after thermal, mechanical, and electrical nociceptive stimulation (Sclocco et al., 2016; Cotton et al., 2018; Courtois et al., 2020). In anesthesia, the HRV measurement is also used in the calculation of the Analgesia-Nociception Index (ANI) in order to control the nociception/anti-nociception balance (De jonckheere et al., 2015). Other studies in healthy subjects or patients able to communicate have also shown an association between HRV and subjective measures of pain such as pain thresholds or pain tolerance (Leźnicka et al., 2017; Paccione et al., 2022). Noteworthy, some studies have failed to find a link between pain stimulation/subjective pain measure and HRV. It has been shown that this physiological marker can also be used as an indication of nociception in patients with DoC. Recent studies found a higher HRV complexity in patients in MCS compared to patients in UWS during nociceptive stimulation (Tobaldini et al., 2018; Riganello et al., 2019). Indeed, a lower HRV complexity index was observed after noxious compared to non-noxious stimulation only in patients in UWS. This decrease in HRV complexity in patients with UWS reflects adaptation difficulties and lower reactivity to nociceptive stimulation (Tobaldini et al., 2018; Riganello et al., 2019; Venturella et al., 2019). In the study by Venturella et al. (2019), nociceptive stimulus processing in patients in UWS was also related to higher delta parietal activation [i.e., involved in attention and perception processing (Güntekin and Başar, 2016)], lower left frontal alpha activation (i.e., left frontal alpha activity related to information inhibition processes), and an increase of galvanic skin response (GSR). These results suggest that nociceptive stimulation can generate a cortical and autonomic response in behaviorally unresponsive patients.

The GSR (also referred to as electrodermal activity or skin conductance) is a biological electrical activity of the skin linked to the activity of the sweat glands which are controlled by the sympathetic system. It is a non-invasive technique allowing the investigation of emotional response following auditory or nociceptive stimulation (Gomez and Danuser, 2004; Khalfa et al., 2008). Studies using the number of skin conductance fluctuations and the normalized skin conductance level in healthy subjects showed that these measures could disentangle noxious stimulation (i.e., heat, mechanical, and cold stimulation) from other sympathetic stimuli (i.e., stimulation by noise and painful images). Indeed, the authors noticed that these measures during noxious stimulation were greater than during other stimulations and correlated with the subjective measure of pain using self-reported pain scale (Günther et al., 2016; Sugimine et al., 2020). Regarding patients with DoC, a study used the GSR and HRV entropy to investigate the autonomic response related to trace conditioning learning in patients in UWS after nociceptive stimulation. Patients in UWS with high GSR showed behavioral signs overlapping with the diagnosis of MCS 4 weeks after the experiment (Cortese et al., 2020). Measurement of GSR to assess the response to nociceptive stimulation during conditional learning may be an additional tool to improve the assessment of patients with DoC.

Finally, the pupillary dilatation reflex (PDR), whose variation results from the balance between the ortho- and parasympathetic tone, represents a promising tool to objectify nociception in DoC. The PDR is used to detect pain in brain-injured patients. It is also sensitive to opioids and allows the assessment of the nociception-anti-nociception balance during general anesthesia (De jonckheere et al., 2015). In the absence of intercurrent factors, PDR may be due to either sympathetic stimulation (e.g., in awake patient) or parasympathetic inhibition (e.g., in anesthetized patient). It is important to note that PDR is also sensitive to tactile stimuli and increased attention/cognitive load or emotional/cognitive arousal (Gusso et al., 2021). This suggests that PDR, related to nociceptive stimulus processing, can be divided into two stages: an excitation stage related to the strength of the stimulus, and an exploration stage related to the emotional processing of the stimulus (Bradley et al., 2008; Gusso et al., 2021). The use of pupillometry to detect the processing of nociceptive stimulus has not been studied in patients with DoC yet. The use of pupillometry to detect the processing of nociceptive stimulus has not been studied in patients with DoC yet. It would be interesting to investigate this topic in future studies, taking care to control for potential confounding factors due to the environment (e.g., change in brightness) or patient’s condition (e.g., presence of eyelid disorder, ptosis or pupil disorder, and mydriasis/myosis).

### 4.2. Treatments for pain

**FIGURE 4:**
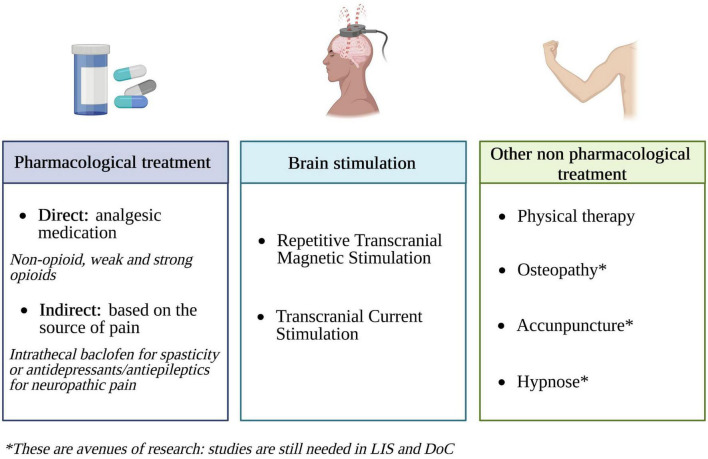
Pain treatment options in DoC and LIS patients (created with BioRender.com). Based on Posadzki and Ernst (2011), Klein et al. (2015), Chatelle et al. (2016), Vickers et al. (2018), Rice et al. (2019), Bicego et al. (2021), and de Pedro Negri et al. (2022).

Pain prevention in patients with DoC still needs improvement. Indeed, a recent pilot clinical trial found that, although the majority of patients showed signs of pain during mobilization, only 33% of them were treated for pain before inclusion in the study (Bonin et al., 2022b). In order to reduce pain in severe brain-damaged patients, both pharmacological and non-pharmacological treatments can be used (Figure 4).

Even if, in clinical practice, the administration of pharmacological treatment is common, it is very important to pay attention to the nature and the dose of these treatments. There are three levels of analgesics: level 1 corresponds to non-opioid medications (e.g., acetaminophen), level 2 to weak opioids (e.g., tramadol) and level 3 to strong opioids (e.g., morphine) (Ventafridda et al., 1985). By preventing the release of acetylcholine in the thalamus, high-doses of opioids may decrease arousal and thus have an impact on the diagnosis as well (Brown et al., 2018). In contrast, the use of an optimal dose of analgesic medications can decrease pain while preserving patients’ level of arousal and consciousness (Chatelle et al., 2016; Lanzillo et al., 2016; Whyte et al., 2020). In an open label study by Chatelle et al. (2016), a decrease in the NCS-R total scores and subscores was observed after analgesic treatment administration (ranging from level 1 to level 3 analgesic medications, depending on patient needs), independently from the diagnosis and etiology. This decrease in NCS-R scores did not lead to a deleterious change in the level of consciousness, with some patients even showing an improvement. Another study showed an increase in the level of consciousness after the administration of an analgesic treatment in patients with DoC who demonstrate severe spastic paresis (Lanzillo et al., 2016). Nonetheless, these results were not replicated in a recent trial by Bonin et al. (2022b) designed to evaluate the effects of analgesic treatment on nociception and pain signs during physiotherapy. This absence of results suggests either a lack of sensitivity of the NCS-R in detecting behavioral changes related to analgesic administration during physiotherapy or a lack of effectiveness of the treatments used. This disparity in outcomes can be related to the fact that Chatelle et al. (2016) conducted an open label research on patients with acute DoC, whereas Bonin et al. (2022b) performed a randomized double-blind placebo-control trial on patients with chronic DoC. Therefore, the lack of improvement in NCS-R scores might be attributed to the ineffectiveness of interventions during the chronic phase or potential bias during the assessment. Indeed, acute and chronic DoC have different pain profiles (i.e., chronic DoC are more prone to develop spastic paresis or neuropathic pain and are thus more resistant to analgesic therapies). Another study performed in a large sample of patients with TBI showed that BINAM scores were also sensitive to the administration of a non-opioid analgesic medication (Whyte et al., 2020). These studies indicate that the use of appropriate analgesia could reduce the risk of misdiagnosis and that the monitoring of pain (i.e., NCS-R and BINAM) as well as arousal/consciousness (i.e., assessed using the CRS-R) is necessary to set a good balance between pain relief and side effects of these treatments. Regarding pain treatment in LIS patients, a recent study highlighted that the majority of the surveyed patients were receiving pain killers (73% non-opioids, 20% non-inflammatory, and 13% weak opioids; Bonin et al., 2022c). In this study, 36% of the surveyed patients were suspected of having neuropathic pain. The first-line treatments for this type of pain are antidepressants and antiepileptics (Foley, 2003). Some of these patients (12%) were indeed being treated with these two types of drugs, but it was not clear from the information collected in the study whether it was given specifically for neuropathic pain or for other reasons. It is also possible to relieve patients’ pain indirectly by acting on the source of the pain. For instance, several studies found beneficial effects of intrathecal baclofen on reducing spastic paresis as well as on improving consciousness recovery (Francois et al., 2001; Shrestha et al., 2011). By decreasing spastic paresis, these approaches could facilitate consciousness recovery by improving motor function and/or reducing pain (Pistoia et al., 2015; Lanzillo et al., 2016).

As explained above, pharmacological treatments often induce side effects that can impact the behavioral responses of patients during evaluations. Therefore, being able to propose non-pharmacological treatments seems essential to manage pain in these patients. The use of invasive brain stimulation techniques such as deep brain stimulation on the PAG and the rostral ventromedial medulla or motor cortex stimulation have proven to be effective in the treatment of chronic pain but remain difficult to implement in patients with DoC (Bittar et al., 2005; Cruccu et al., 2007; Lima and Fregni, 2008; Fontaine et al., 2009). Although less effective than invasive stimulation, a possible alternative to these methods would be the use of non-invasive stimulation techniques such as repetitive transcranial magnetic stimulation or transcranial direct current stimulation (Klein et al., 2015; Lefaucheur et al., 2017). The effectiveness of physiotherapy or aerobic exercises (in combination with other methods) has also shown beneficial effects for pain management in LIS patients (Rice et al., 2019). Regarding other non-pharmacological approaches, and according to Bonin et al.’s (2022c) survey, only a minority of LIS patients have ever tried methods such as osteopathy, acupuncture, or electromagnetic therapy and none have tried hypnosis, relaxation, or meditation. None of these methods have been specifically investigated in patients with LIS, while some techniques could be of particular interest for these patients. Although used in the clinical setting on other pathologies, some of the methods are still controversial. Osteopathy, for instance, shows different results depending on the type of pain. A systematic review investigating osteopathy on musculoskeletal pain did not provide convincing evidence of efficacy in treating such pain (Posadzki and Ernst, 2011). However, another systematic review focusing on chronic low back pain found osteopathy to be effective in relieving it (Dal Farra et al., 2021). A recent meta-analysis showed that acupuncture can be effective in some cases of chronic pain such as musculoskeletal, headache, and osteoarthritis pain (Vickers et al., 2018). A study in mice also showed the effectiveness of this technique in relieving allodynia and improving emotional/cognitive dysfunction caused by neuropathic pain (Jang et al., 2021). Regarding electromagnetic therapy, systematic reviews of patients with musculoskeletal or chronic pelvic pain have shown that this method could be effective, but further studies are needed to examine the use of more standardized protocols (Paolucci et al., 2020; de Pedro Negri et al., 2022). Studies focusing on the use of hypnosis in healthy subjects and patients with acute or chronic pain highlighted a modulation of pain perception during the hypnotic state (Rainville, 1997; Vanhaudenhuyse et al., 2018; Bicego et al., 2021). A multiple-case study found that self-hypnosis could also be a useful tool to improve the quality of life of patients suffering from phantom limb pain (i.e., sensation of pain in a limb that has been amputated) by reducing the intensity of the pain, whether sensory or affective (Bicego et al., 2022). The reduction of pain sensation induced by hypnosis allows decreasing the doses of analgesics usually administered to these patients and thus improves their level of arousal and quality of life. The use of this technique in LIS patients, by avoiding side effects such as fatigue, could allow them to make the most of their communication tools. Meditation is an approach that has not yet been studied in LIS patients. However, experts in meditation show a decrease in pain sensitivity associated with an increase in brain activity in regions involved in pain processing, and a decrease in brain activity in regions involved in emotional processing and executive functions (Grant et al., 2011; Gard et al., 2012). It is hypothesized that the decrease in cognitive and emotional processing of the nociceptive stimulus may facilitate the association of the noxious stimulus with a neutral rather than unpleasant valence.

## 5. Reflections and future directions

Regarding the assessment of pain and nociception in patients with DoC or LIS, there are currently no clear guidelines and no clinical consensus. When performing neuroimaging analyses, it is relevant to mention that differences in terms of structures and physiological properties may exist between a severe brain-injured patient and a healthy subject. Therefore, it is essential to perform a multimodal assessment, not only based on neuroimaging but also on pain-related behaviors and physiological changes [(i.e., increase of heart rate and respiratory rhythm, and skin conductance (Cowen et al., 2015; Devalle et al., 2018; Riganello et al., 2019)] to improve pain assessment and indirectly the diagnosis of these patients. From a behavioral perspective, opinions still differ among researchers and clinicians regarding some behaviors that could be reflective of cortical processing (Poulsen et al., 2019). This is particularly the case for facial expression such as grimacing and crying. Indeed, even if grimacing is considered as an indicator of pain, the Multi-Society Task Force on Permanent Vegetative State does not consider it as a necessary sign of conscious perception, as it can occur reflexively through subcortical pathways in the thalamus and limbic system (The Multi-Society Task Force on PVS, 1994). Patients showing no sign of consciousness except for grimaces to nociceptive stimuli can therefore be diagnosed as being in UWS. Moreover, some patients in LIS suffer from cortical lesions. This impacts their cognitive functions by impairing, for example, the recognition of negative facial expressions, or leading to the development of pathological laughter and crying which may distort the assessment of pain (Leonard et al., 2019). Many pain scales take into account the assessment of facial expressions in a more or less developed way (Feldt, 2000; Gélinas et al., 2006; Chanques et al., 2009; Chatelle et al., 2012). However, the facial expression assessment is clinically scored based on gross observation of facial movements in response to a noxious stimulation. A better characterization of facial expressions could be an interesting avenue of research to improve the behavioral assessment of these patients. For instance, the use of the facial action coding system could be developed in these patients. This system allows the coding of different types of emotions (including pain) based on the anatomical analysis of facial movements. It can distinguish 46 different action units produced by a single muscle or a combination of muscles (Kunz et al., 2007, 2008; Bartlett et al., 2014).

Numerous studies have highlighted the relevance of measuring neurophysiological parameters in the assessment of pain and nociception (Riganello et al., 2019; Cortese et al., 2020). At present, very few studies have investigated the clinical utility of GSR and PDR in the assessment of pain in DoC. This is mainly due to the fact that these measures are not suitable for all types of DoC patients, some of whom may suffer from ptosis often associated with the presence of myosis (i.e., pupil constriction) or other pupillary reactivity disorders, which makes it difficult to measure PDR. In addition, it is important to note that there is a gap between research and practice. The scientific literature on LEPs is well developed, but in practice, this technique is more complicated to implement in a systematic way. The device allowing LEP measurement is an expensive non-portable system, difficult to use in a clinical setting, especially with a sensitive population such as patients with DoC. Other less costly and easier to use techniques assessing the integrity of the spinothalamic pathways are used in other populations and deserve to be investigated in patients with DoC and LIS. For instance, pinprick-evoked potentials (PEPs, mechanical stimulation) are useful to assess the functional integrity of mechano-nociceptive pathways and detect central sensitization (Iannetti et al., 2013; Rosner et al., 2020; van den Broeke et al., 2020) but could be difficult to use in non-collaborative population such as DoC patients. Then, cool-evoked potentials (CEPs, thermal stimulation) allow the evaluation of the integrity of the spinothalamic pathways by stimulating Aδ-fibers and participate in the diagnosis of neuropathic pain without inducing pain (De Keyser et al., 2018; Leone et al., 2019). Finally, contact heat-evoked potentials (CHEPs, thermal stimulation) are also used to specifically assess the nociceptive component of a stimulus. These new generation of thermal cutaneous stimulators (i.e., thermodes) are portable and easier alternatives to LEPs for the recording of robust nociceptive (heat) and non-nociceptive (cold) responses in patients with DoC (De Schoenmacker et al., 2021; Lejeune et al., 2022). The aforementioned techniques could allow better understanding of nociception processing and facilitate neuropathic pain detection in patients with DoC and LIS, which is currently understudied. In the future, the NCS-R could be improved by integrating new physiological parameters like other recently developed scales, such as the BINAM for TBI patients or the PAS. Moreover, the measurement of physiological parameters could facilitate the assessment of the nociception/anti-nociception balance after analgesic administration. Indeed, to monitor the effects of analgesics administered during general anesthesia, anesthesiologists can use different types of tools measuring the activity of the autonomic nervous system (De jonckheere et al., 2015). The above-mentioned ANI, for instance, is based on HRV analysis and allows the measurement of the relative parasympathetic tone. Its score ranges from 0 to 100, a low score meaning that the patient is able to process nociceptive stimulus. The Surgical Pleth Index (SPI) is rather based on the measurement of the orthosympathetic hemodynamic response to noxious stimulation, and uses normalized heartbeat intervals (HBIs) and plethysmography wave amplitude for its calculation (Rogobete et al., 2021). The PDR and the GSR are also used in anesthesia to assess the sympathetic tone but have not yet been studied in detail in patients with DoC and LIS. The functional near-infrared spectroscopy (fNIRS) applied to pain detection could also be an avenue of future research to investigate. It is a non-invasive, low cost, easy-to-use, and portable brain imaging technique that allows to measure cortical hemoglobin concentration changes (Barati et al., 2017; Lopez-Martinez et al., 2019). Studies in healthy subjects have shown that fNIRS can provide an objective and robust assessment of pain by measuring changes in hemoglobin in the sensorimotor and prefrontal cortex (Yücel et al., 2015). Its application for pain detection has also been studied in sensitive and non-communicative patient populations such as infants and critically ill patients (Ranger and Gélinas, 2014; Yuan et al., 2022). The fNIRS is also used in patient with DoC to improve diagnosis but there is not, to our knowledge, any study specifically related to the detection of pain in this population (Rupawala et al., 2018). This measurement technique could also be an avenue to develop in post-coma patients given its low cost, ease of use and portability.

In the future, it would also be essential to develop non-pharmacological therapies in order to limit the use of analgesics and thus avoid the side effects such as fatigue or decreased vigilance. Few studies have looked at the effects of music therapy on the level of consciousness of UWS and MCS patients and have shown that it is a safe and effective method that can improve functional outcomes of patients [for a meta-analyses refer to Li et al. (2020)]. The effect of this technique on pain perception in LIS and DoC patients has not, to our knowledge, been studied yet. However, studies carried out in other patient populations have shown interesting effects (by reducing anxiety for instance) which suggest that this may be an interesting avenue to investigate in future research (Lin et al., 2020; Santiváñez-Acosta et al., 2020; Dallı et al., 2022; Seyffert et al., 2022).

This review focuses mainly on the physical pain that DoC and LIS patients may experience. However, LIS patients may also suffer from emotional pain such as depression or anxiety (Bergés et al., 2007; Rousseau et al., 2015; Bonin et al., 2022c). More studies are still needed to better characterize this type of suffering and its impact on patients’ daily lives in order to propose appropriate pharmacological (e.g., antidepressants and anxiolytics) and complementary (e.g., hypnosis and meditation) treatments.

## 6. Conclusion

There are still many unknowns in the assessment, management and treatment of pain in DoC and LIS patients. The NCS-R remains the most appropriate way to assess pain in patients with DoC but could be improved by considering the inclusion of physiological parameters in their behavioral assessment. The measurement of pain and nociception should be done with a multimodal approach, also taking into account (neuro)physiological and neuroimaging data as complementary measures. It is known that some behavioral UWS patients may show preservation of cortical areas involved in nociceptive signal processing. Then, pain assessment and analgesic treatments should be applied in a more systematic way, and most importantly, independently of patient’s clinical diagnosis. In particular, titration of analgesic agents should be implemented to determine the optimal dose of the medications. The NCS-R and the BINAM represent relevant assessment tools to find a balance between reduced pain and preserved level of consciousness following analgesic treatment. For the moment, the guidelines of the American Academy of Neurology recommend the use of the NCS-R to assess pain in patient with DoC but these guidelines still need to be developed further and refined (Giacino et al., 2018). Regarding patients in LIS, even if they do not communicate their pain spontaneously, it is important to actively and regularly make an assessment through the use of simple communication codes. When signs of pain are detected, it is essential to identify the source of the physical and emotional pain to be able to propose appropriate treatments, both pharmacological and non-pharmacological.

## Author contributions

EB: conceptualization, research, references formatting, writing, and editing. AT: conceptualization, supervision, review and editing, visualization, and resources. NL, ES, VB, CM, OG, and SL: review and resources. All authors contributed to the article and approved the submitted version.
