# Supplementary material for: Assessment and management of pain/nociception in patients with disorders of consciousness or locked-in syndrome: A narrative review
Source: Front Syst Neurosci. 2023 Mar 20;17:1112206. doi: 10.3389/fnsys.2023.1112206 (PMC10067681; doi:10.3389/fnsys.2023.1112206)
Supplement: Supplementary file 1 [file Table_1.DOCX]

**Supplementary Table 1: Comparison of pain and nociception assessment scales in DoC patients.**

| **Scales** | **Characteristics** | **Stimulation and scoring** | **Advantages** | **Limitations** |
| --- | --- | --- | --- | --- |
| **Coma Recovery Scale-Revised (CRS-R,** (67)**)** | Assess the level of consciousness but motor subscales include an assessment of pain response. | - Deep pressure on the nailbed of the finger/toe (2 trials on each side on the body) for a minimum of 5 seconds. | / | - Scale developed to determine the patient's altered state of consciousness. - Takes into account only the motor response to pain. |
| **Nociception Coma Scale Revised**  **(NCS-R,** (93)**)** | - 5-minute observation. - Three subscales, nine items: - Motor response: localization to painful stimulation, flexion withdrawal, abnormal posturing. - Verbal response: verbalisation (intelligible), vocalisation, groaning. - Facial expression: cry, grimace, oral reflexive movement/startle response. | - Patient's responses are observed at rest (1 minute of observation) and during a potentially painful care (e.g., physical therapy) or stimulation (e.g., pressure on the nailbed of the finger/toe). - All observed behaviors are scored. The total score (ranging from 0 to 9) is the sum of the highest subscore of each subscale. - Score of 2 specific to nociception. - Score ≥ 5 related to cortical pain processing. | - Good concurrent validity and inter-rater reliability. - Significant difference between noxious (acute pain) to non-noxious stimulus (tactile). - Sensitive to pain-inducing conditions (e.g., physical therapy). - Validation study used acute standardized stimulus. | - Facial expression not detailed enough. - Dependent on clinical diagnosis (correlation with CRS-R). - Does not include physiological parameters. - Lack of clear guidelines based on recent studies. |
| **Pain Assessment Scales**  **(PAS,** (110)**)** | - Four subscales, 27 items:  1. *Physiological/autonomic*: blood pressure, heart rate, respiratory rate, pupillary dilatation, tear production, pale skin, cold skin, cold sweating, sweat production, goose pimples. 2. *Body language:* grimaces, forehead wrinkles, squinting, clenched teeth, eyes wide open, increased tonus, twitches. 3. *Verbal communication:* sigh, moan, complain, cry. 4. *Behavior:* weeps, irritability, motor restlessness, confusion, restlessness, takes hand to the aching place. | - Assessment before and after pain-relieving procedures (repositioning in bed and administration of analgesics). - Items rated as “present” or “not present”. | - Very good interrater reliability for 13/27 items and good interrater reliability for 8/27 items. - Good sensitivity to change after repositioning. - Assess behavioral and physiological parameters. | - Preliminary results. - No use of standardized stimulus. - Some items with moderate interrater reliability (pale skin, moan and grimacing). - Does not detect changes after antalgic administration. |
| **Brain Injury Nociception Assessment Measure (BINAM,** (111)**)** | - 15-minute observation. - 10 items: arousal, diastolic blood pressure, heart rate, systolic blood pressure, pupil size, vocalization, movement, posture, facial expression, skin color. | - Assessment after repositioning in bed, after repositioning in a chair or wheelchair, in bed or in a wheelchair after prolonged sitting/lying, and during physical therapy. - Each item has a specific ranking from 2 to 7. | - Scores are independent of the diagnosis and state of agitation. - Assess behavioral and physiological parameters. - Sensitive to pain-inducing conditions (physiotherapy) and antalgic treatment. | - Preliminary results. - No use of standardized stimulus. - No psychometric values available. |
